# Ferumoxtran-10-enhanced MRI for pre-operative metastatic lymph node detection in pancreatic, duodenal, or periampullary adenocarcinoma

**DOI:** 10.1007/s00330-024-10838-w

**Published:** 2024-06-22

**Authors:** Geke Litjens, Atsushi Nakamoto, Lodewijk A. A. Brosens, Marnix C. Maas, Tom W. J. Scheenen, Patrik Zámecnik, Erwin J. M. van Geenen, Mathias Prokop, Kees J. H. M. van Laarhoven, John J. Hermans

**Affiliations:** 1 Department of Medical Imaging, Radboud Institute for Health Sciences, Radboud University Medical Center, Nijmegen, The Netherlands; 2 Department of Radiology, Osaka University Graduate School of Medicine, Suita, Japan; 3 Department of Pathology, Radboud University Medical Center, Nijmegen, University Medical Center Utrecht, Utrecht, The Netherlands; 4 Department of Gastroenterology and Hepatology, Radboud Institute for Molecular Life Sciences, Radboud University Medical Center, Nijmegen, The Netherlands; 5 Department of Surgery, Radboud Institute for Health Sciences, Radboud University Medical Center, Nijmegen, The Netherlands

**Keywords:** Pancreatic cancer, Periampullary cancer, Duodenal cancer, Magnetic resonance imaging, Lymph node metastasis

## Abstract

**Objectives:**

To assess 3-Tesla (3-T) ultra-small superparamagnetic iron oxide (USPIO)-enhanced MRI in detecting lymph node (LN) metastases for resectable adenocarcinomas of the pancreas, duodenum, or periampullary region in a node-to-node validation against histopathology.

**Methods:**

Twenty-seven consecutive patients with a resectable pancreatic, duodenal, or periampullary adenocarcinoma were enrolled in this prospective single expert centre study. Ferumoxtran-10-enhanced 3-T MRI was performed pre-surgery. LNs found on MRI were scored for suspicion of metastasis by two expert radiologists using a dedicated scoring system. Node-to-node matching from in vivo MRI to histopathology was performed using a post-operative ex vivo 7-T MRI of the resection specimen. Sensitivity and specificity were calculated using crosstabs.

**Results:**

Eighteen out of 27 patients (median age 65 years, 11 men) were included in the final analysis (pre-surgery withdrawal *n* = 4, not resected because of unexpected metastases peroperatively *n* = 2, and excluded because of inadequate contrast-agent uptake *n* = 3). On MRI 453 LNs with a median size of 4.0 mm were detected, of which 58 (13%) were classified as suspicious. At histopathology 385 LNs with a median size of 5.0 mm were found, of which 45 (12%) were metastatic. For 55 LNs node-to-node matching was possible. Analysis of these 55 matched LNs, resulted in a sensitivity and specificity of 83% (95% CI: 36–100%) and 92% (95% CI: 80–98%), respectively.

**Conclusion:**

USPIO-enhanced MRI is a promising technique to preoperatively detect and localise LN metastases in patients with pancreatic, duodenal, or periampullary adenocarcinoma.

**Clinical relevance statement:**

Detection of (distant) LN metastases with USPIO-enhanced MRI could be used to determine a personalised treatment strategy that could involve neoadjuvant or palliative chemotherapy, guided resection of distant LNs, or targeted radiotherapy.

**Registration:**

The study was registered on clinicaltrials.gov NCT04311047. https://clinicaltrials.gov/ct2/show/NCT04311047?term=lymph+node&cond=Pancreatic+Cancer&cntry=NL&draw=2&rank=1.

**Key Points:**

- *LN metastases of pancreatic, duodenal, or periampullary adenocarcinoma cannot be reliably detected with current imaging*.
- *This technique detected LN metastases with a sensitivity and specificity of 83% and 92%, respectively*.
- *MRI with ferumoxtran-10 is a promising technique to improve preoperative staging in these cancers*.

## Introduction

Lymph node (LN) metastases are associated with decreased overall survival (OS) for pancreatic ductal adenocarcinoma (PDAC, 11% vs 4% 5-year OS), duodenal, and periampullary tumours (cholangiocarcinoma 47% vs 24% 5-year OS, duodenal cancer 65% vs 21% 5-year OS, and ampulla of Vater cancer 61 vs 25 months median survival) [1–4]. Especially para-aortic LN metastases, classified as distant metastases by the TNM criteria of the Union for International Cancer Control (UICC, [5]), are associated with lower survival [6, 7]. Therefore, adequate LN assessment is critical in managing these cancers.

Conventional diagnostic imaging for pancreatic, duodenal, and periampullary cancer, including contrast-enhanced computed tomography (CECT), magnetic resonance imaging (MRI), fluorine-18-fluorodeoxyglucose positron emission tomography-computed tomography ([^18^F]FDG-PET-CT), and endoscopic ultrasound (EUS), demonstrate limited sensitivity and specificity for detecting LN metastases. CECT for pre-operative assessment of extra-regional LN metastases in pancreatic, duodenal, and periampullary cancer has a sensitivity of 25–29% and a specificity of 83–86% [8, 9]. A study focusing on para-aortic LN metastases in pancreatic cancer with CECT, MRI, and [^18^F]FDG-PET-CT failed to identify any metastatic nodes, resulting in a sensitivity of 0% for all modalities [10]. Although only six patients in this study had histologically proven para-aortic LN metastases, this underscores the challenges in the accurate detection of metastases in this region. Additionally, a study involving 490 patients with resected PDAC found no significant difference in the percentage of LN metastases at histopathology between patients with or without enlarged LNs on preoperative imaging (CT, MRI, or EUS) [11]. This study concluded that size, commonly relied upon for LN metastase detection, is unreliable in predicting malignant LNs.

Ferumoxtran-10, an ultra-small superparamagnetic iron oxide (USPIO) particle, is a potentially valuable MRI contrast agent for detecting LN metastases [12, 13]. After intravenous administration, macrophages take up these particles and accumulate in non-metastatic, i.e. healthy (parts of) LNs. The paramagnetic properties of the iron core locally disturb the homogeneity of the MR system’s main magnetic field. Therefore, healthy LNs, with accumulated USPIO particles, exhibit a reduced signal intensity on T2*-weighted sequences. Conversely, in LN areas with metastases, the absence of USPIO particles results in a (partly) high signal intensity on T2*-weighted sequences [14]. A meta-analysis from 2011 combining different cancer types showed a mean sensitivity of 90% and a specificity of 96% of USPIO-enhanced MRI in detecting malignant LNs [15]. However, no studies with pancreatic, duodenal, or periampullary adenocarcinoma were included and results from tumours with a different entity, anatomical location, and vascularisation cannot simply be extrapolated.

We hypothesise that USPIO-enhanced MRI can accurately detect LN metastases for pancreatic, duodenal, and periampullary tumours. Accurate identification of LN metastases could tailor treatment plans including palliative or neoadjuvant chemotherapy, targeted resection of distant LNs, or targeted radiotherapy. This study aims to assess 3-T USPIO-enhanced MRI in detecting LN metastases for resectable adenocarcinomas of the pancreas, duodenum, or periampullary region, in a node-to-node validation against histopathology.

## Materials and methods

The institutional review board approved this prospective single-centre cohort study and written informed consent was obtained. The study was registered on clinicaltrials.gov (NCT04311047) and conducted in accordance with the Declaration of Helsinki and the Dutch Medical Research Involving Human Subjects Act. Treatment-naïve patients with pancreatic, duodenal, or periampullary adenocarcinoma scheduled for surgical resection at the Radboudumc (tertiary hepatobiliary centre) between May 2017 and December 2020 were consecutively included. Eligibility was determined at the multidisciplinary pancreaticobiliary cancer meeting. The periampullary region was defined as the distal common bile duct, ampulla of Vater, and 2 cm of the duodenum surrounding the ampulla. Exclusion criteria were neoadjuvant treatment, distant metastases on preoperative imaging, other concomitant malignancies (prior malignancies: at least 5 years disease-free), or contraindications for MRI or USPIO (allergy, hemochromatosis, thalassemia, or sickle cell anaemia).

### Procedures

Preoperative USPIO-enhanced MRI was conducted using ferumoxtran-10 (Ferrotran, SPL Medical B.V., investigational product), administered intravenously (2.6 mg/kg body weight) 24–36 h before the scan, as previously described [16]. MRI was performed on a 3-T MRI system (Magnetom Prisma, Siemens Healthcare). Butylscopolamine and glucagon were administered to minimise peristaltic motion, unless contraindicated. Patients were positioned feet first supine, with body phased array coils around the upper abdomen. The scan range was from diaphragm to aortic bifurcation. Images were acquired with repeated breath-holds of maximally 20 s on expiration. MRI sequences included T2-weighted HASTE for anatomical reference, T1-weighted VIBE DIXON for LN localisation, and fat-suppressed T2*-weighted multi gradient echo (mGRE) sequence for USPIO visualisation, mGRE images were reconstructed to a single T2*-weighted computed echo time (TE) of 12 ms [17]. Table 1 describes the technical details of the MR sequences.

**Table 1:** Technical details of the MR sequences on 3-T MRI

Sequence	T1-VIBE DIXON		T2-HASTE		T2*-weighted mGRE	
Orientation	Coronal	Axial	Coronal	Axial	Coronal	Axial
Acquisition mode	3D breathhold		2D multiple breathhold		3D multiple breathhold	
Resolution (mm^3^)	1.4 × 1.4 × 1.7	1.3 × 1.3 × 3.0	1.6 × 1.6 × 5.0	1.2 × 1.2 × 5.0	1.4 × 1.4 × 1.4	1.3 × 1.3 × 1.3
FOV (mm^2^)	394 × 450	306 × 380	400 × 400	309 × 380	265 × 313	210 × 291
Slices/partitions per BH	104	64	10	11	20	20
Number of BH	1	2	4	4	4	16
Acquired TE (ms)	1.23, 2.46		87	95	2.7–16.7	
Reconstructed TE (ms)	NA		NA		12	
TR (ms)	4.21	3.90	NA		20.0	
Bandwidth (Hz/Pix)	1040	1020	698	710	380	
Flip angle (°)	9		156	160	10	
Fat suppression	None		None		FatSat	

*3D* 3-dimensional, *FatSat* spectral fat saturation, *FOV* field of view, *ms* milliseconds, *BH* breathhold, *TE* echo time, *TR* repetition time, *VIBE* volumetric interpolated breath-hold examination, *mGRE* multi gradient echo, *NA* not applicable

### Image evaluation

A radiologist (A.N., 18 years experience in abdominal imaging) analysed all USPIO-enhanced MRI images. A second radiologist (J.H., 23 years experience in pancreatic imaging) supervised the analysis. Disagreements were resolved in a consensus meeting. Both radiologists were blinded to the histopathology results. All visible LNs, regional and distant, were annotated and measured (short axis on axial orientation). LN locations were indicated using the classification of the Japan Pancreas Society [18]. LNs were scored on the iron-sensitive T2*-weighted mGRE (TE = 12 ms) sequence, using diagnostic guidelines adapted from Anzai et al (Table 2, [19]). Type 1–4 LNs were classified as suspicious for metastases and type 5–7 LNs as non-suspicious. The distinction between regional and distant LNs was based on the TNM classification (eighth edition) by the UICC [5]. The definition of regional nodes differs depending on the cancer type (pancreatic head or tail, cholangial, ampullary, or duodenal), the respective definition for each tumour type was applied.

**Table 2:** Diagnostic guidelines used for scoring LNs on MRI (adapted from Anzai et al [19])

LN type	LN signal post-USPIO	Description	Diagnosis
1		No blackening of node or node is hyperintense to surrounding tissue	Suspicious
2		Node has a central high signal with darkening along the peripheral rim	Suspicious
3		Partial darkening whereby more than 50% of the node has a high signal intensity	Suspicious
4		Less than 50% of nodes have a high signal intensity	Suspicious
5		Node having an overall dark signal other than a central or hilar area of fat seen on T1 sequence	Non-suspicious
6		Node having an overall dark signal with speckles of subtle granularities	Non-suspicious
7		Node having an overall dark signal intensity	Non-suspicious

### Surgical resection and specimen analysis

Operative procedures, performed by experienced surgeons in pairs (mean experience nine years), contained pylorus resecting pancreatoduodenectomy (PRPD) with lymphadenectomy (stations no. 5, 6, 8a, 12b1/2, 12c, 13a/b, 14a/b, and 17a/b) or distal pancreatectomy (DP) with lymphadenectomy (station no. 10, 11, and 18) as indicated by the International Study Group on Pancreatic Surgery (ISGPS) [20]. Additional para-aortic or other distant LNs were resected if indicated as suspicious on MRI and resection was technically possible and safe. Resection specimens were pinned on an anatomical drawing for orientation.

A high-resolution ex vivo MRI of the fresh resection specimen and separately resected LNs was performed on a 7-T preclinical MRI system (Clinscan, Bruker® BioSpin). Ex vivo images had a resolution of 0.29 × 0.29 × 0.29 mm^3^ and consisted of a water and lipid-excited T1-weighted image according to a predefined workflow [16]. Subsequently, the tumour was fixated in formaldehyde for at least 48 h and then sliced into 4-mm slices following the axial slicing technique [21]. LNs from the peripancreatic fat were removed and enclosed separately, LNs larger than 5 mm were sectioned in 2–3 mm thick sections for paraffin embedding. Coupes were 4 µm with haematoxylin and eosin (H&E) staining. In contrast to measurement on MRI (short axis on axial orientation), at pathology, the largest diameter of the LNs was measured, as is clinical routine.

### Node-to-node evaluation

Aided by the ex vivo MRI, LNs from in vivo MRI were matched to pathology for node-to-node analysis. Clinical follow-up imaging was used to confirm if an LN was indeed resected or not. Matching criteria were size, shape, and location, including anatomical landmarks such as surrounding vessels and organs. Only matched LNs were included in the validation analysis. A pancreas pathologist (L.B., ten years of expertise) re-evaluated all matched LNs.

Statistical analysis was performed using SPSS (version 25). The sensitivity and specificity of USPIO-MRI were calculated with crosstabs, using pathology as the reference standard. Continuous variables were summarised using standard descriptive statistics (mean, standard deviation, median, and range), and categorical variables were summarised with frequencies.

### Re-evaluation

After prospective analysis, a third radiologist (P.Z., eight years experience with USPIO-enhanced MRI) reviewed all false positive and false negative LNs to investigate potential reasons for misclassification.

## Results

**Fig. 1:**
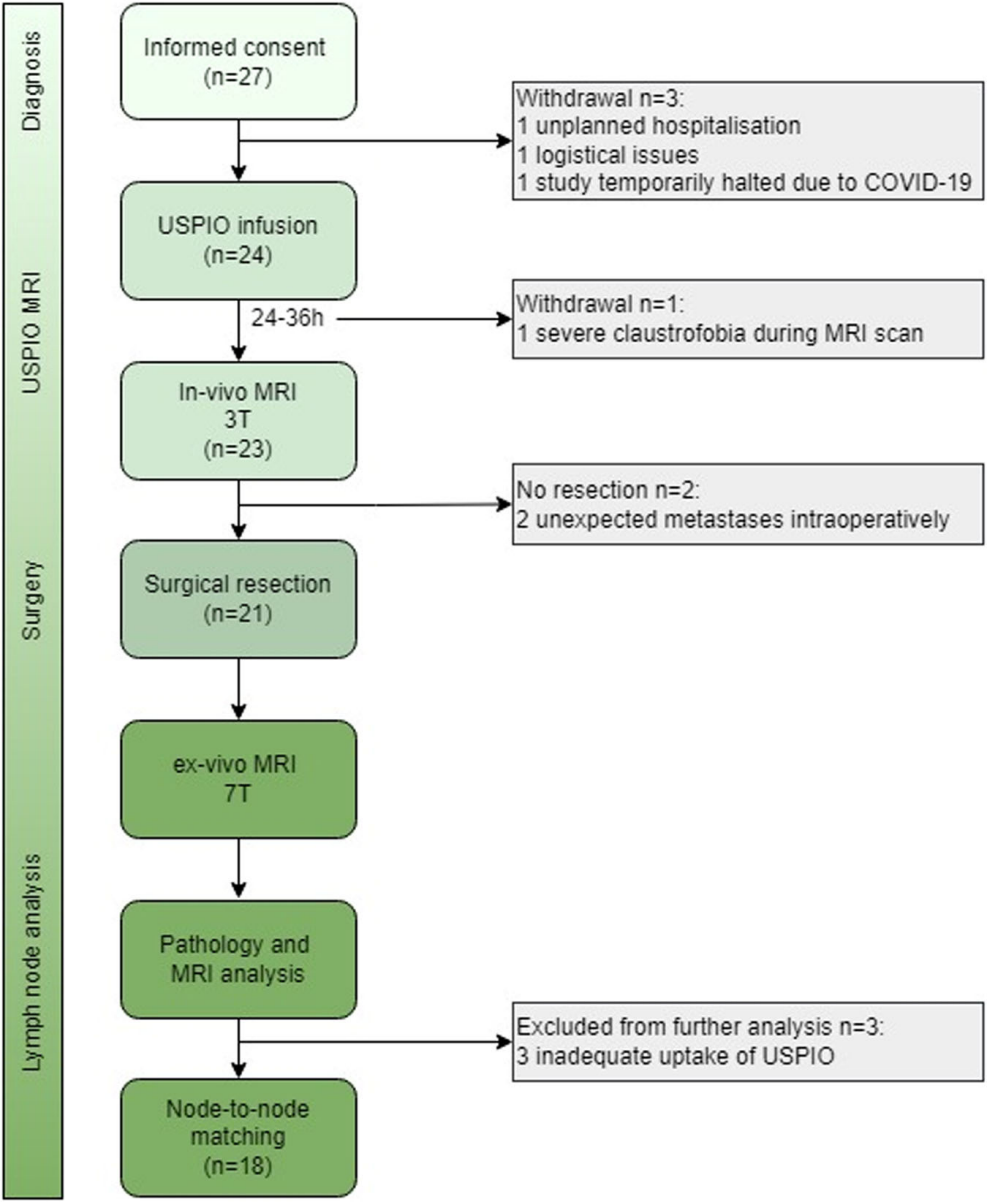
Flowchart of patient inclusion, study procedures, and analysis

Twenty-seven treatment-naïve patients scheduled for resection were enrolled in this prospective study. Figure 1 shows the patient flow. Four patients were excluded before surgery, and two patients were excluded because they were not resected due to unexpected metastases found intraoperatively. In three patients all LNs, both supra- and infra-diaphragmatic, had a high signal intensity on T2*-weighted images, suggesting inadequate uptake or distribution of ferumoxtran-10. Patient characteristics (age, tumour type, medical history, BMI, smoking, and alcohol), laboratory results (bilirubin, renal function, haemoglobin, and leucocytes), MRI analysis (no other abnormalities were seen), and histopathological investigation (both metastatic and normal benign LNs were present in each patient at histopathology), did not show a possible cause for non-uptake of ferumoxtran-10. We excluded these three patients from further analysis. Eighteen patients (11 male, seven female; median age 65) were included in the final analysis (characteristics in Table 3). Supplementary Fig. S1 and S2 show the distribution of regional and distant LNs and size distribution. The time between MRI and surgery was seven days median (range 1–26). At least one follow-up CT was performed in 13 patients (72%), the first CT was after 3 months median (range 1–13).

**Table 3:** Patient and tumour characteristics of patients included in the final analysis

Patient	Sex	Age, (years)	Tumour type	Resection method	TNM	Resection margin	Total nr of LNs at pathology, (regional/distant)	Malignant LNs at pathology, (regional/distant)	Total nr of LNs on MRI, (regional/distant)	Suspicious LNs on MRI, (regional/distant)
1	M	62	Cholangiocarcinoma	PRPD	pT3N0M0	R0	48 (46/2)	0 (0/0)	42 (9/33)	2 (0/2)
2	F	79	PDAC (pancreatic head)	PRPD	pT3N2M1^a^	R1	23 (22/1)	11 (10/1)	19 (4/15)	2 (0/2)
3	F	67	PDAC (pancreatic head)	PRPD	pT2N1M0	R1	28 (18/10)	3 (3/0)	9 (0/9)	5 (0/5)
4	M	61	PDAC (pancreatic tail)	DP	pT3N0M0	R1	20 (20/0)	0 (0/0)	42 (3/39)	0 (0/0)
5	M	55	Cholangiocarcinoma	PRPD	pT2N1M0	R1	21 (14/7)	1 (1/0)	28 (9/19)	3 (2/1)
6	M	65	Ampulla of Vater (intestinal type)	PRPD	pT2N0M0	R0	20 (17/3)	0 (0/0)	23 (9/14)	5 (0/5)
7	F	71	Ampulla of Vater (pancreaticobiliary type)	PRPD	pT3N1M0	R0	18 (18/0)	1 (1/0)	34 (6/28)	1 (0/1)
8	M	58	Cholangiocarcinoma	PRPD	pT2N2M1^a^	R1	29 (27/2)	9 (7/2)	41 (5/36)	22 (2/20)
9	M	53	Duodenal carcinoma	PRPD	pT4N1M0	R1	11 (11/0)	2 (2/0)	5 (3/2)	0 (0/0)
10	F	53	Cholangiocarcinoma	PRPD	pT3N0M0	R0	15 (14/1)	0 (0/0)	16 (6/10)	0 (0/0)
11	M	65	Cholangiocarcinoma	PRPD	pT3N2M0	R0	13 (12/1)	2 (2/0)	22 (8/14)	3 (1/2)
12	F	80	Cholangiocarcinoma	PRPD	pT2N1M1^a^	R1	18 (17/1)	11 (10/1)	12 (4/8)	1 (0/1)
13	M	72	Duodenal carcinoma	PRPD	pT3N1M0	R0	18 (18/0)	1 (1/0)	45 (13/32)	1 (1/0)
14	F	61	Ampulla of Vater (pancreaticobiliary type)	PRPD	pT2N0M0	R0	14 (14/0)	2 (2/0)	14 (6/8)	2 (1/1)
15	M	81	Cholangiocarcinoma	PRPD	pT2N0M0	R0	11 (9/2)	0 (0/0)	19 (7/12)	1 (0/1)
16	M	74	Cholangiocarcinoma	PRPD	pT3N1M0	R0	22 (22/0)	0 (0/0)	12 (3/9)	0 (0/0)
17	M	65	Ampulla of Vater (pancreaticobiliary type)	PRPD	pT2N1M0	R0	13 (12/1)	1 (1/0)	45 (8/37)	8 (0/8)
18	F	70	Ampulla of Vater (pancreaticobiliary type)	PRPD	pT3N1M0	R0	43 (35/8)	1 (1/0)	25 (7/18)	1 (1/0)

*M* male, *F* female, *PRPD* pylorus resecting pancreatoduodenectomy, *DP* distal pancreatectomy

^a^M1 due to distant LN metastases at histopathology

### Safety

Ferumoxtran-10 administration and MRI were performed according to protocol in 23 patients, without any adverse events. Additional para-aortic LNs were resected for study purposes in 11 patients. Postoperative chyle leak occurred in three patients of which only one had undergone additional para-aortic LN resection.

### LN scoring USPIO-enhanced MRI

In the 18 patients included in the final analysis, 453 LNs in total were detected on the USPIO-enhanced MRI. Their median size was 4.0 mm (range 2–20 mm) and there was a median of 23 LNs per patient (range 5–45). The majority of LNs were distant (*n* = 343). Assessment of the second radiologist and consensus meeting resulted in eight additionally identified LNs, four classified as not being LNs, ten LNs with a change in score without a change in suspicion, and ten LNs with a change in score that also resulted in a change of suspicion (four from non-suspicious to suspicious and six from suspicious to non-suspicious).

Fifty-eight LNs (13%) were scored as suspicious (type 1–4) on mGRE (TE = 12 ms), 394 LNs (87%) as non-suspicious (type 5–7), and one LN was not evaluable. The number of LNs for each type is shown in Table 4. Twenty-seven LNs (6%) were 10 mm or larger on MRI, of which 11 (41%) were scored as suspicious. Most suspicious LNs were para-aortic for all tumour types, except duodenal adenocarcinoma (zero suspicious para-aortic LNs).

**Table 4:** LN scoring on MRI according to the diagnostic guidelines described in Table 2

LN type	Frequency, *n* (%)	Diagnosis
1	43 (9.5%)	Suspicious
2	4 (0.9%)	Suspicious
3	5 (1.1%)	Suspicious
4	6 (1.3%)	Suspicious
5	0 (0%)	Non-suspicious
6	4 (0.9%)	Non-suspicious
7	390 (86.1%)	Non-suspicious
Not evaluable	1 (0.2%)	Not evaluable

### Histopathology

At histopathology, 385 LNs in total were found, the median size was 5.0 mm (range 1–27 mm) and there was a median number of 19 LNs per patient (range 11–48). The majority of LNs were regional (*n* = 346). Forty-five LNs (11.7%) were metastatic, of which 41 were regional, and four were distant (resulting in M1 classification in patient nr. 2, 8, and 12). Seventy-five LNs (19%) were 10 mm or larger, of which 15 (20%) were metastatic.

### Node-to-node correlation

Node-to-node matching from USPIO-MRI to histopathology was possible for 55 LNs from the 18 included patients. Six of these LNs were metastatic and 49 benign. Median size was 5.0 mm on MRI (range 2–14 mm) and 10.0 mm at histopathology (range 3–27 mm). Most matched LNs were regional LNs (*n* = 46). There were five (9%) true positives, 45 (82%) true negatives, four (7%) false positives, and one (2%) false negatives on mGRE TE = 12 ms. Sensitivity and specificity on a node-to-node basis for mGRE TE = 12 ms were 83% (95% CI: 36–100%) and 92% (95% CI: 80–98%), respectively.

### Re-evaluation

**Fig. 2:**
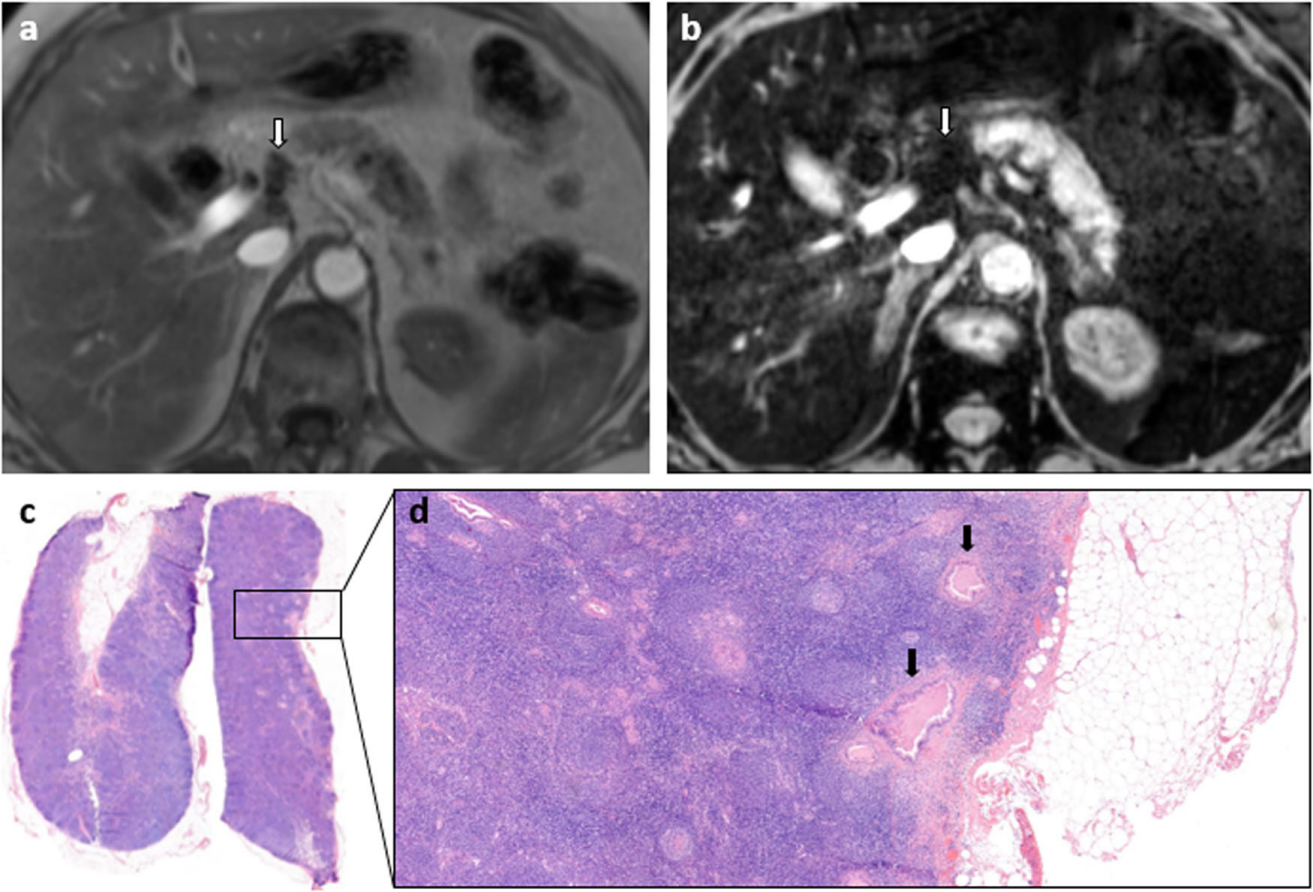
An example of a false negative LN from an 80-year-old female patient with cholangiocarcinoma, not suspected on USPIO-MRI, but metastatic at histopathology. **a** T1-VIBE in phase, axial view; **b** mGRE TE = 12 ms (iron sensitive), axial view; **c** malignant LN at histopathology, H&E staining; and (**d**) magnified part of malignant LN with small malignant depositions (black arrows) visible in an abundance of normal lymphatic tissue. The LN is indicated with white arrows on the MRI images and shows strong signal loss on the iron-sensitive sequence (type 7)

**Fig. 3:**
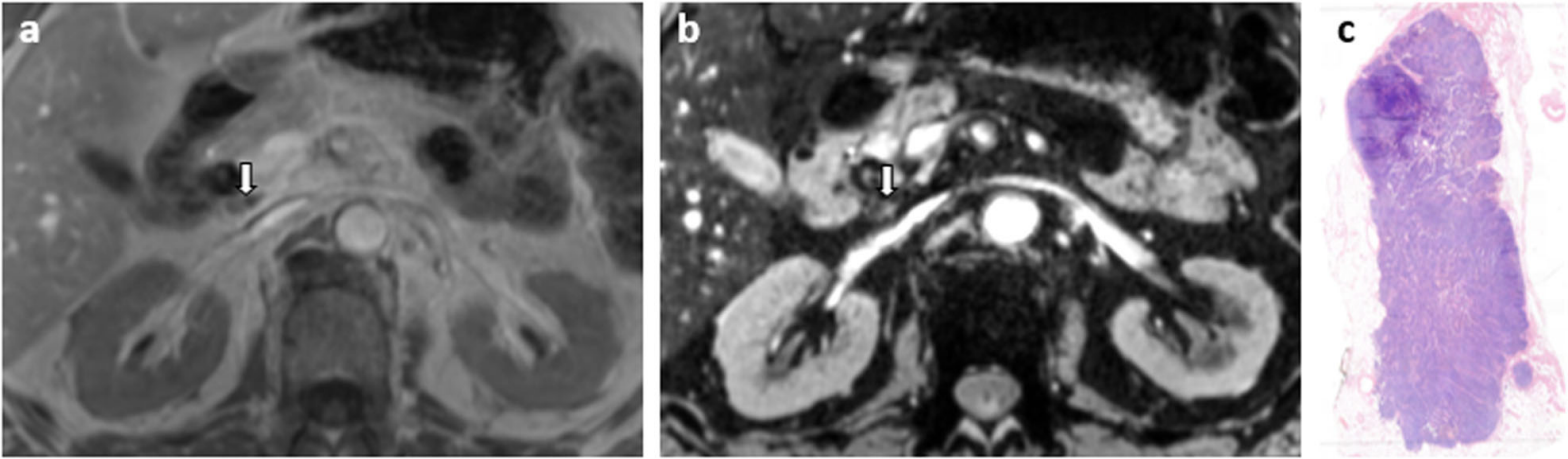
An example of a false positive LN from an 81-year-old male patient with cholangiocarcinoma, suspect on USPIO-MRI (type 1) but negative at histopathology. **a** T1-VIBE in phase, axial view, LN indicated with the white arrow; **b** mGRE TE = 12 ms (iron sensitive), axial view, LN indicated with the white arrow, partly shows a high signal intensity, and is thus indicated as suspicious; and (**c**) benign LN at histopathology, H&E staining

**Fig. 4:**
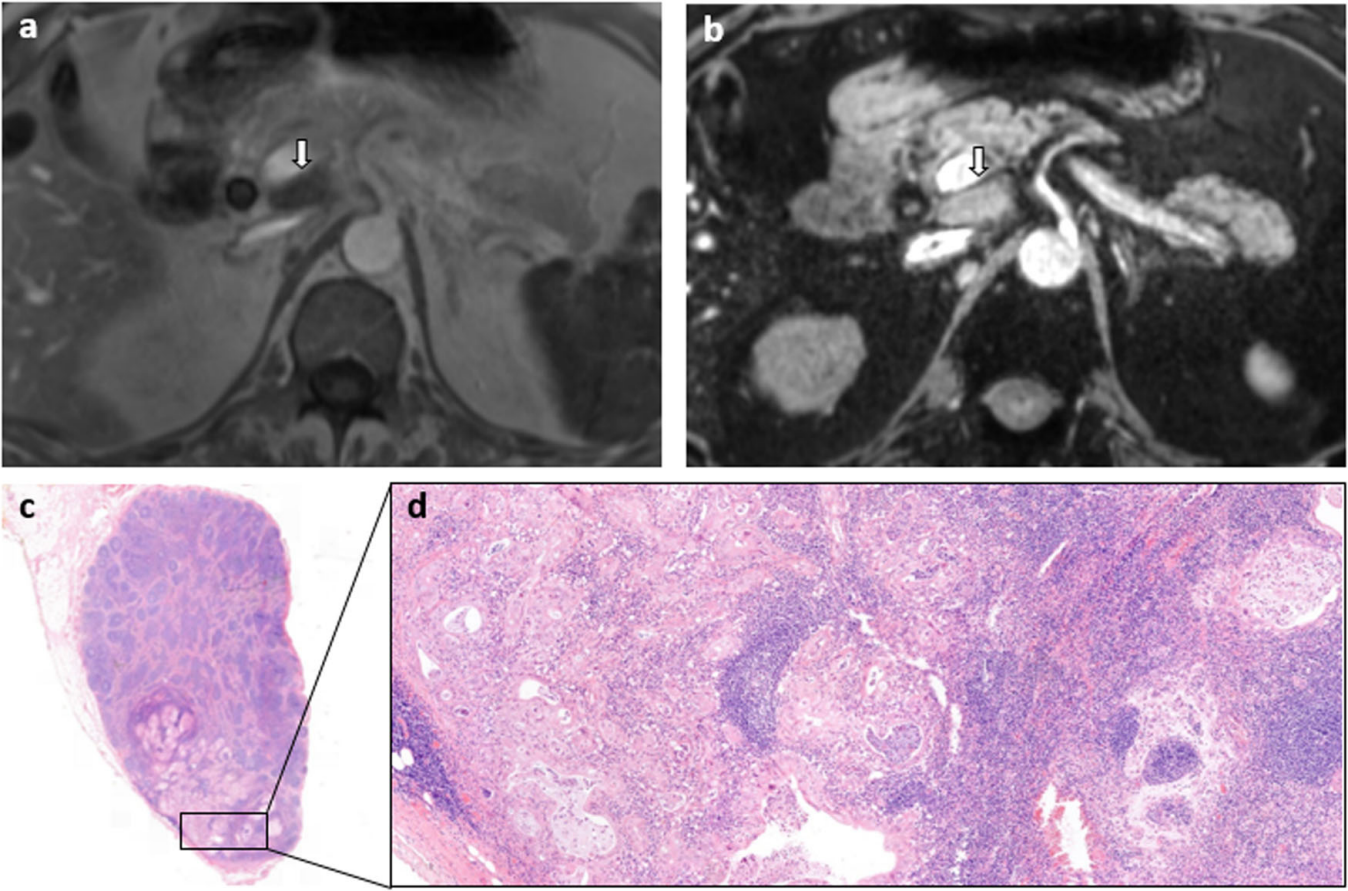
An example of a true positive LN from a 58-year-old male patient with cholangiocarcinoma, suspect on USPIO-MRI (type 1) and metastatic at histopathology. **a** T1-VIBE in phase, axial view, LN indicated with the white arrow; **b** mGRE TE = 12 ms (iron sensitive), axial view, LN indicated with the white arrow; **c** malignant LN at histopathology with metastatic tissue in the entire LN, H&E staining; and (**d**) magnified part of malignant LN with malignant cells visible

**Fig. 5:**
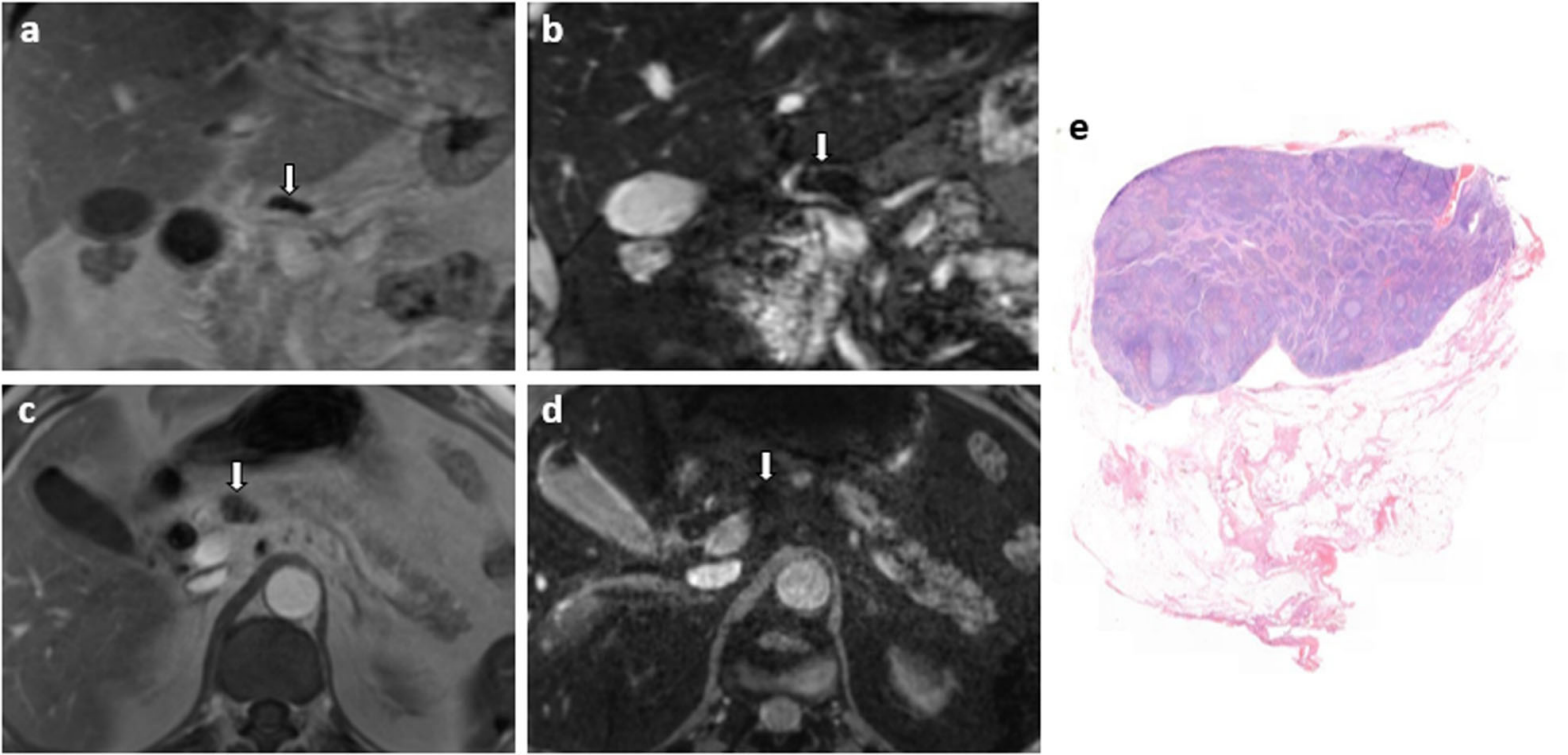
An example of a true negative LN from a 55-year-old male patient with cholangiocarcinoma, not suspect on USPIO-MRI (type 7) and negative at histopathology. **a** T1-VIBE in phase, coronal view, LN indicated with the white arrow; **b** mGRE TE = 12 ms (iron sensitive), coronal view, LN indicated with the white arrow; **c** T1-VIBE in phase, axial view, LN indicated with the white arrow; **d** mGRE TE = 12 ms (iron sensitive), axial view, LN indicated with the white arrow; and (**e**) benign LN at histopathology, H&E staining

The false negative (*n* = 1) and false positive (*n* = 4) LNs were re-evaluated. The false negative LN showed an area of 9 mm with multiple small metastatic depositions at histopathology, within an abundance of normal LN tissue with probably a normal iron uptake causing strong signal loss on MRI (Fig. 2). The first false positive LN showed homogeneous intermediate elevated signal intensity on T2*-weighted images, suggesting moderately reduced iron uptake, falsely interpreted as metastatic. The reason for this reduction was unclear. The second false positive LN remained suspect upon re-evaluation, with high signal intensity (Fig. 3). Even after thorough pathology examination, no metastases were found in these two cases. The two other false positives were caused by adjacent structures with high signal intensity being misinterpreted as LNs (small vessels and ganglions). Figures 4 and 5 show an example of a true positive and true negative LN, respectively.

## Discussion

This prospective study presents a node-to-node validation of in vivo 3-T MRI with ferumoxtran-10 against histopathology for the detection of LN metastases in patients with resectable pancreatic, duodenal, or periampullary adenocarcinoma. USPIO-enhanced T2*-weighted mGRE reached a node-to-node sensitivity of 83% and specificity of 92%.

Only one previous study investigated USPIO-enhanced MRI in pancreatic cancer, using a different nanoparticle (ferumoxytol), reporting a patient-level sensitivity of 83% and specificity of 80%, based on 11 patients of which seven had PDAC. However, this study did not perform node-to-node matching and lacked clarity on the inclusion of para-aortic LNs [22]. We chose to perform node-to-node analysis with the matching of individual LNs, for detection and localisation of LN metastasis.

Our node-to-node analysis results were comparable to previous studies on LN metastase detection using USPIO-enhanced MRI in other cancer types. For instance, a sensitivity of 70% and specificity of 98% was found in node-to-node analysis for axillary LNs in 16 breast cancer patients [23]. A node-to-node study on rectal cancer demonstrated true negative rates ranging from 82% to 86% in ten patients, five of which were however partially treated with neo-adjuvant therapy [24]. A study of 77 patients with multiple cancer types found a sensitivity of 87–93% and a specificity of 73–85% [25]. Like the present study, these three studies evaluated LNs based on criteria proposed by Anzai et al [19]. A recent study in head and neck cancer introduced a novel reading algorithm, comparing LN signal intensity to lipid tissue intensity, instead of evaluating the LNs own signal pattern. Histopathologically malignant LNs showed an equal or higher signal intensity compared to lipid tissue [26]. Future research should explore the applicability of a similar strategy in pancreatic, duodenal, and periampullary cancer.

Despite our well-defined workflow [16], node-to-node correlation of in vivo MRI with histopathology was possible for only 55 LNs. This was partly due to the large number of distant LNs on MRI outside the surgical field, which could not be safely resected. Additionally, numerous small LNs directly adjacent to the pancreas, duodenum, or tumour identified at pathology, were often missed on MRI. Despite the fact that suspect LNs on MRI were shown to the surgeon preoperatively, not all distant LNs were resected, proven by their continued presence on follow-up imaging. LNs near the left renal vein were more easily identified and resected during surgery compared to those situated between the aorta and vena cava, as those are located too dorsally for safe resection. Finally, anatomical matching of LNs found on MRI within the surgical field still proved to be challenging, despite the use of ex vivo MRI, due to specimen shrinkage and altered orientation after fixation.

The majority of LNs on MRI was < 10 mm, a commonly used cut-off size for benign vs metastatic LNs [27] the majority of suspicious LNs (47/58 = 81%) on MRI was also < 10 mm. At histopathology only 20% (15/75) of LNs > 10 mm were metastatic. This suggests that LNs < 10 mm may be metastatic more frequently than commonly assumed and supports that size is not an adequate tool to detect LN metastases [11].

In our study, para-aortic LNs were the most frequently identified suspicious LNs on MRI for all tumour types, except for duodenal adenocarcinoma. Para-aortic LN metastases are regarded as distant metastases (M1) according to the TNM classification [5]. Nevertheless, the implications of distant LN metastases on treatment remains a subject of ongoing debate. This is illustrated by two meta-analyses from 2016 which included nearly identical studies but reported contradictory recommendations about resection of the primary tumour in the presence of para-aortic LN metastases [6, 7]. Extended resection of distant LNs is not recommended according to consensus reached in 2014 by the International Study Group on Pancreatic Cancer [20]. It does not improve survival and is associated with increased postoperative complications, such as chyle leakage [28–30]. However, studies show that adjuvant chemotherapy or chemoradiotherapy can be beneficial in patients with LN metastases in the resection specimen [31, 32]. One study showed adjuvant chemoradiotherapy has an additional survival effect compared to adjuvant chemotherapy alone, for patients with LN metastases [31]. Furthermore, in a retrospective study of patients with cN1 pancreatic cancer who received neoadjuvant chemotherapy, 38% were downstaged to ypN0 with improved survival [33]. Finally, lower rates of metastatic LNs at histopathology are reported in patients treated with neoadjuvant chemoradiotherapy compared to patients receiving immediate surgery [34]. When radiotherapy was involved, these studies used large elective radiotherapy treatment fields to cover all potentially involved LNs and to compensate for treatment uncertainties like motion, causing considerable toxicity. Given the limitations of currently used diagnostic imaging in detecting LN metastases and the uncertainty of the exact implications of the presence of LN metastases, there is still inconsistency when formulating a treatment plan. As our study shows that USPIO-enhanced MRI may be a relevant imaging technique for the detection and localisation of LN metastases, it would provide an understanding of the pattern of LN metastases and has the potential to improve therapy in patients with pancreatic, duodenal, or periampullary adenocarcinoma. Current MR-guided stereotactic body radiation therapy may play a role in this strategy by allowing the treatment of isolated LN metastases with high accuracy and limited toxicity [35]. Further studies are needed to prove this hypothesis.

Our study has several limitations. We had to exclude three patients due to unexplained inadequate uptake or distribution of USPIO contrast. Despite extensive analysis, no specific cause was identified. Therefore, for future USPIO studies, we recommend assessing the adequate contrast agent uptake in LNs first, also using sLNs in other anatomical areas for quality control. Additionally, an extensive resection of distant LNs for correlation with histopathology was not performed, because this would be unsafe due to the increased risk of complications. Finally, different measurements for LN size on MRI and pathology had to be used, making size comparison difficult. These different measurements are the result of different slicing planes on MRI and histopathology—it is technically very difficult to make them identical.

## Conclusion

Concluding, in this study we performed node-to-node correlation of USPIO-enhanced MRI to histopathology in patients with pancreatic, duodenal, or peri-ampullary adenocarcinoma. The results show that USPIO-enhanced MRI is a promising technique to preoperatively detect LN metastases in patients with pancreatic, duodenal, or periampullary adenocarcinoma, with a high sensitivity and specificity. This holds promise for early detection and localisation of both local and distant LN metastases, which can be used to improve staging and guide neoadjuvant and adjuvant therapy, such as targeted radiotherapy. Further studies are needed to reveal the therapeutic potential of using USPIO-enhanced MRI for therapy guidance.

## Supplementary information

Electronic Supplementary Material

## Abbreviations

CECT: Contrast-enhanced computed tomography
EUS: Endoscopic ultrasound
^18^FDG-PET-CT: Fluorine-18-fluorodeoxyglucose positron emission tomography-computed tomography
H&E: Haematoxylin and eosin
LN: Lymph node
mGRE: Multi gradient echo
MRI: Magnetic resonance imaging
OS: Overall survival
PDAC: Pancreatic ductal adenocarcinoma
UICC: Union for International Cancer Control
USPIO: Ultra-small superparamagnetic iron oxide
