# Supplementary material for: Ferumoxtran-10-enhanced MRI for pre-operative metastatic lymph node detection in pancreatic, duodenal, or periampullary adenocarcinoma
Source: Eur Radiol. 2024 Jun 22;34(12):7973–84. doi: 10.1007/s00330-024-10838-w (PMC11557713; doi:10.1007/s00330-024-10838-w)
Supplement: Supplementary file 1 — Electronic Supplementary Material [file 330_2024_10838_MOESM1_ESM.pdf]

Supplementary material

Supplement to: Ferumoxtran-10-enhanced MRI for pre-operative metastatic lymph node detection in pancreatic, duodenal, or periampullary adenocarcinoma.

Lymph node distribution

The distribution of the number of regional and distant LNs on MRI and at histopathology per patient is shown in Figure S1. The distribution of LN size of the non-suspicious and suspicious LNs on MRI and at histopathology is shown in Figure S2.

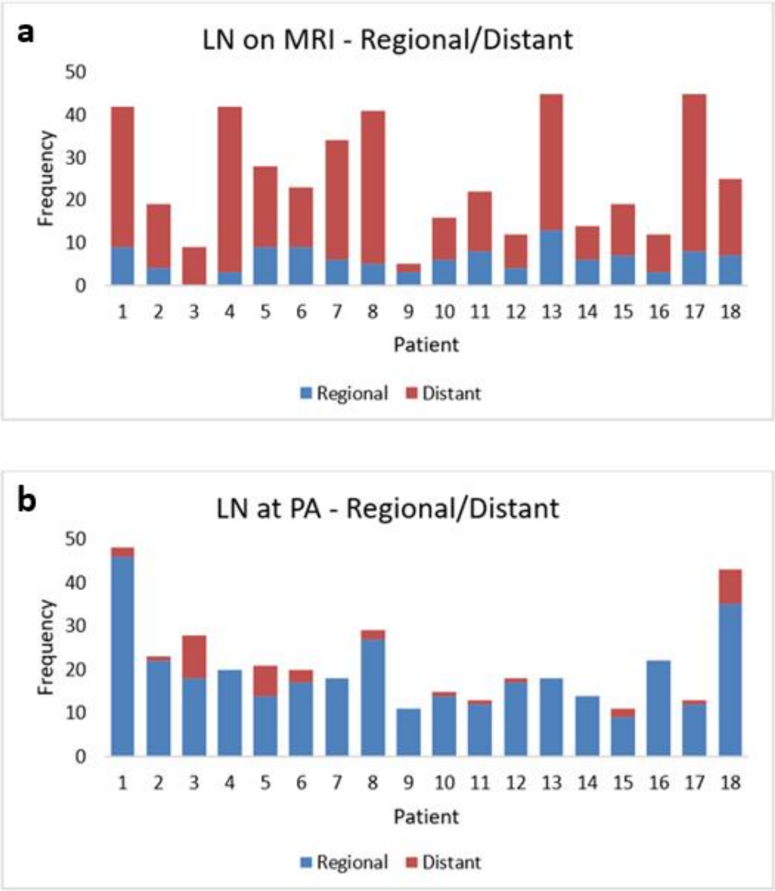

Figure S1: a: Distribution of number of regional and distant lymph nodes detected on USPIO-enhanced MRI per patient. Total LNs: median 23, range 5-45. Regional LNs: median 6, range 0-13. Distant LNs: median 15, range 2-39. **b:** Distribution of number of regional and distant lymph nodes found at histopathology per patient. Total LNs: median 19, range 11-48. Regional LNs: median 18, range 9-46. Distant LNs: median 1, range 0-10

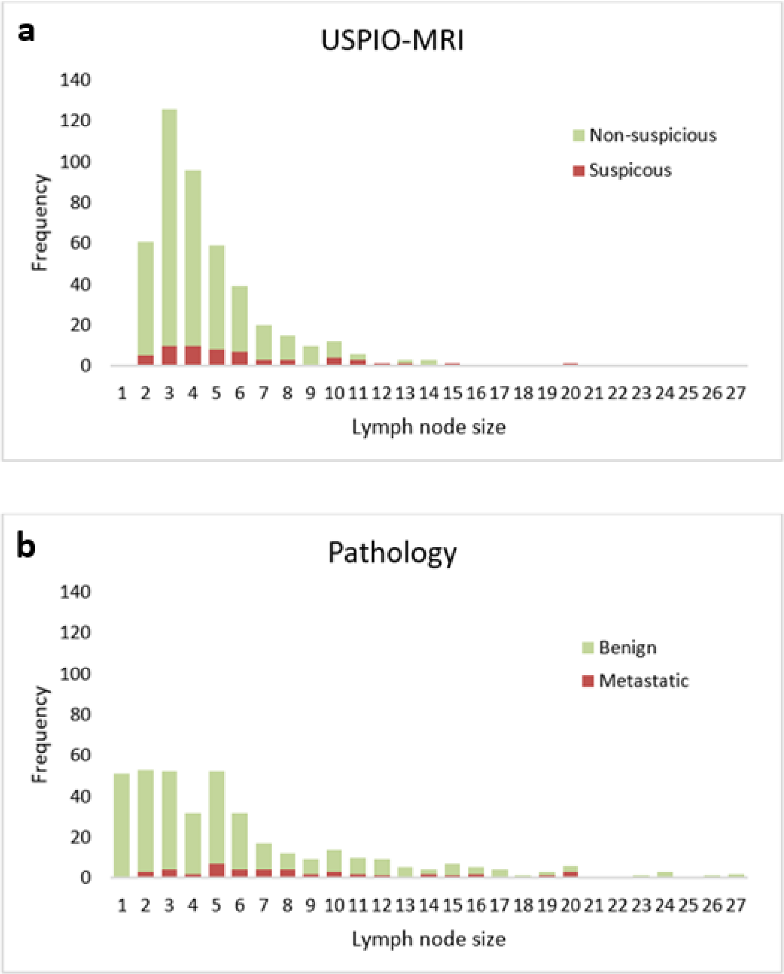

Figure S2: Size distribution of all detected lymph nodes on MRI and at histopathology, divided into non-suspicious/benign (green) and suspicious/metastatic (red). **a:** distribution on USPIO-MRI (shortest axis measured on axial orientation). Out of 453 total LNs, 394 (87%) were non-suspicious, 58 (13%) were suspicious, and 1 (0.22%) was non-evaluable. Out of 426 LNs <10mm, 47 (11%) were suspicious, while out of 27 LNs ≥10mm, 11 (41%) were suspicious. **b:** distribution at histopathology (largest diameter measured on H&E). Out of 385 total LNs, 340 (88%) were benign and 45 (12%) LNs were metastatic.
